# Challenges of Implementing the 4Ms in Federally Qualified Health Centers and Private Academic Medical Centers

**DOI:** 10.1093/geroni/igaa057.2959

**Published:** 2020-12-16

**Authors:** Bonnie Olsen, Theresa Sivers-Teixeira, Kelly Sadamitsu

**Affiliations:** Keck School of Medicine of USC, Alhambra, California, United States

## Abstract

As our country increases our capacity to provide quality interprofessional geriatric medical care to older adults, we find challenges that are unique to the setting. Many older adults receive care in Federally Qualified Healthcare Centers where implementing Age-Friendly Care is critical. Implementing the 4M’s in this setting has specific challenges including lack of geriatric trained staff, staff turnover, leadership engagement and financial sustainability. When implementing Age-Friendly Care within a large-scale complex academic health system, equally difficult challenges surface. With less nimble infrastructures, changes in one service area can have a domino effect and create larger barriers in other parts of the system. Instituting the 4M’s in an academic health system environment requires a careful strategic approach and support from many areas of organizational leadership. This talk will focus on strategies to anticipate and adapt implementation plans to best address barriers that are unique to the setting.

